# Supplementary material for: The interactome of intact mitochondria by cross-linking mass spectrometry provides evidence for coexisting respiratory supercomplexes
Source: Mol Cell Proteomics. 2017 Dec 8;17(2):216–32. doi: 10.1074/mcp.RA117.000470 (PMC5795388; doi:10.1074/mcp.RA117.000470)
Supplement: Supplemental Data [file supp_RA117.000470_133922_0_supp_23978_fzffwf.zip › spectra_annotation/mito_spectra_annotation/384-1-1-1-7-1.pdf]

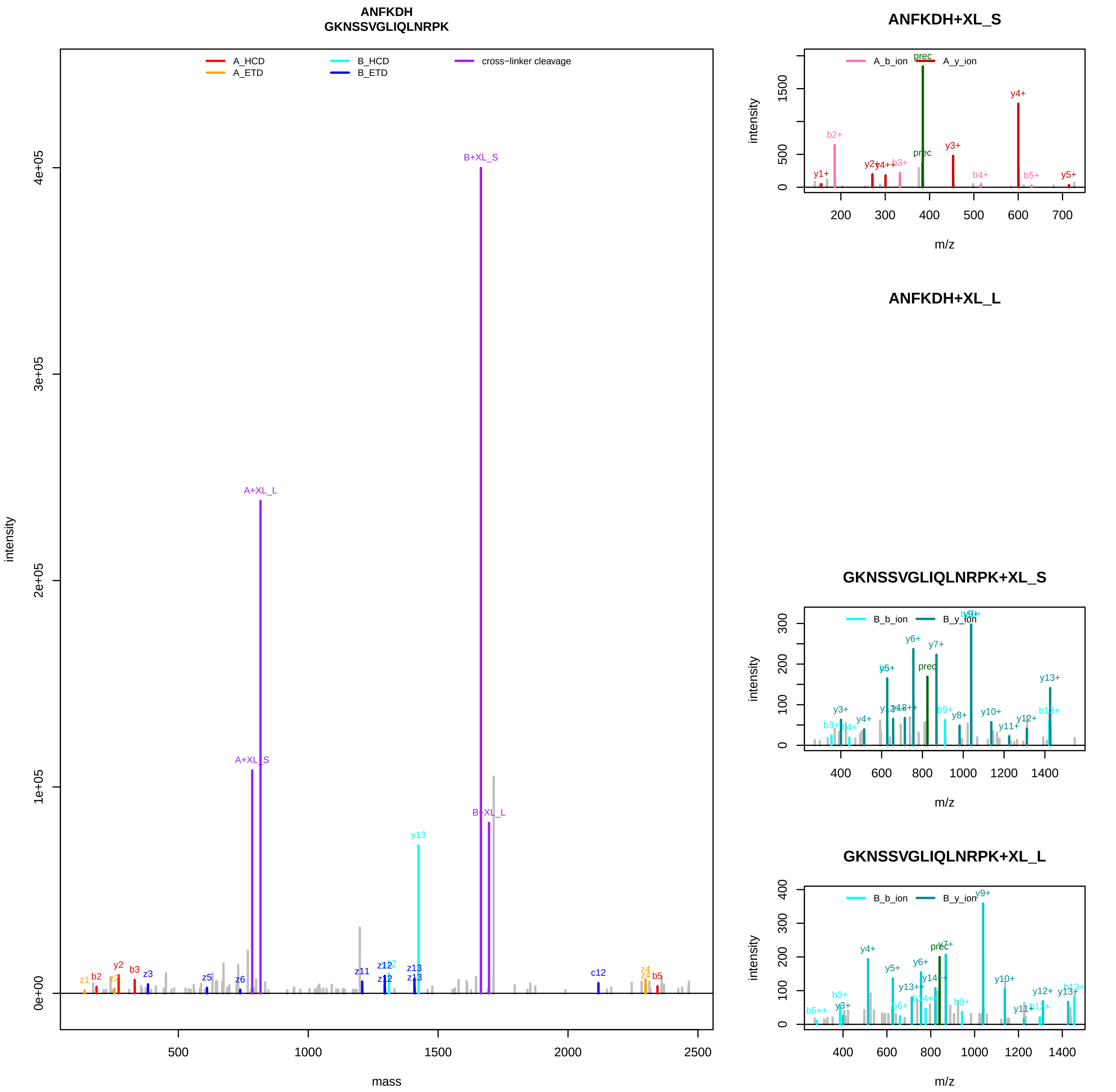
ANFKDH
GKNSSVGLIQLNRPK
A_HCD
A_ETD
B_HCD
B_ETD
cross−linker cleavage
intensity
mass
0e+00
1e+05
2e+05
3e+05
4e+05
500
1000
1500
2000
2500
B+XL_S
A+XL_L
A+XL_S
B+XL_L
y13
z1
b2
z2
y2
b3
z3
z5
z6
z11
z12
z13
c12
z4
b5
ANFKDH+XL_S
A_b_ion
A_y_ion
prec
b2+
y1+
y2+
y4++
b3+
y3+
b4+
y4+
b5+
y5+
m/z
ANFKDH+XL_L
GKNSSVGLIQLNRPK+XL_S
B_b_ion
B_y_ion
y3+
b3+
y4+
y5+
y6+
y7+
y8+
y9+
y10+
y11+
y12+
y13+
b13+
GKNSSVGLIQLNRPK+XL_L
y4+
y5+
y6+
y7+
y9+
y10+
y12+
y13+
b5++
b3+
b6+
b9+
b13+
